# Influence of age and sex on the relative biological effectiveness of 13-keV/μm carbon ions for lifespan shortening of B6C3F1 Mice

**DOI:** 10.1371/journal.pone.0332270

**Published:** 2025-09-23

**Authors:** Shizuko Kakinuma, Yi Shang, Yoshiko Amasaki, Shinobu Hirano-Sakairi, Tomoko Sawai, Takamitsu Morioka, Kazuhiro Daino, Benjamin J. Blyth, Mayumi Nishimura, Masaaki Sunaoshi, Chizuru Tsuruoka, Tatsuhiko Imaoka, Yoshiya Shimada

**Affiliations:** 1 Department of Radiation Effects Research, Institute for Radiological Science, National Institutes for Quantum Science and Technology (QST), Japan; 2 Radiobiology for Children’s Health Program, Research Center for Radiation Protection, National Institute of Radiological Sciences, Chiba, Japan; Central Research Institute of Electric Power Industry (CRIEPI), JAPAN

## Abstract

Epidemiological studies of Japanese atomic bomb survivors indicate that the risk of cancer from radiation exposure is higher in individuals who are relatively young at the time of exposure, with women facing a more significant risk compared to men. However, this type of data is limited for other radiation types, such as particle radiations. Low linear energy transfer (LET) carbon ions are a type of particle radiation to which humans may be exposed as cosmic radiation during long-duration space missions and as radiation passing through healthy tissue during carbon ion radiotherapy. This raises concerns about the risk of late complications, including cancer development. To address these issues, we examined the lifespan of mice after exposure to γ rays or low-LET carbon-ion beams, assessed the effects of sex and age at the time of exposure, and calculated the RBE. Male and female B6C3F1 mice of various ages (embryonic days 3, 13, and 17, and postnatal weeks 1, 3, 7, and 15) were whole-body irradiated a single time with ^137^Cs γ rays (662 keV) or 290-MeV/u monoenergetic carbon ions (LET, ~ 13 keV/µm), and their lifespan was analyzed. For both γ rays and carbon ions, the hazard ratio for mortality increased in a dose-dependent manner, was higher for females than for males, and peaked at 1 week of age at the time of exposure. The RBE of low-LET carbon ions for lifespan shortening was 0.9–1.8 for females and 1.2–2.0 for males, regardless of the age at exposure. Thus, the risk associated with low-LET carbon ion exposure varied with age and sex, but RBE did not. These findings provide essential data for assessing the impacts of low-LET carbon ion exposure.

## Introduction

Epidemiological studies of Japanese atomic-bomb survivors and post-radiotherapy patients have shown that radiation exposure increases the risk of death from cancer and late-occurring noncancer diseases (e.g., circulatory and respiratory) [1–3]. In general, the risk of cancer is greater than that of noncancer diseases, higher in people who are relatively young at the time of exposure, and higher in women than men. However, this type of data is limited regarding other radiation qualities, such as particle radiation.

Carbon ions are a type of particle radiation to which humans may be exposed during space travels, missions, and carbon-ion radiotherapy. Space radiation, which includes protons and ions of helium, carbon, and iron, raises concerns about delayed adverse effects and shortened lifespans for astronauts due to continuous exposure during long-duration space missions [4]. Carbon ion radiotherapy enables precise local dose distribution due to its sharp Bragg peak and high linear energy transfer (LET). The LET range of carbon ions in tumors is 40–90 keV/µm, with relative biological effectiveness (RBE) values for tumor cell killing ranging between 2 and 3 [5,6]. Effective local control of tumors is thus achieved. While the LET of carbon ions in the healthy tissue in front of the tumor is as low as 13 keV/µm [7], there is concern about the risk of developing late-occurring complications, including second cancers and other non-cancer diseases, after carbon-ion radiotherapy, especially in children, because carbon ions produce more numerous clustered DNA-damage sites than photons [8,9].

Dosimetry estimates and retrospective studies have been conducted to predict second cancers and other late-occurring complications. A dosimetry study using a Monte Carlo simulation revealed that the carbon-ion dose delivered to healthy tissue in front of the tumor is lower than the dose within the tumor [10], and the risk of a second cancer of any type is predicted to be comparable between carbon-ion and proton radiotherapies [11,12]. Furthermore, retrospective cohort studies have indicated that the risk of second cancers and adverse effects after carbon-ion radiotherapy in patients with localized prostate cancer and cervical cancer (mostly aged over 40 years) is either lower or comparable to that after photon radiotherapy [13–15]. Nevertheless, concerns persist regarding the risk of second cancer and other late effects in younger patients due to the absence of RBE data on life-threatening late complications in healthy tissues and the limited number of young patients receiving the treatment, which complicates risk estimation.

Epidemiological studies also suggest that the lack of information on lifestyle factors (e.g., diet, smoking history) among subjects may confound the estimation of risk [16,17]. On the other hand, animal studies facilitate the evaluation of the effects of radiation exposure without such confounders. The lifespan shortening of animals exposed to whole-body irradiation has been regarded as a simple and reliable means of estimating the risk of death from all causes [18,19]. Indeed, analyses of lifespan shortening in RFM and BALB/c mice exposed to γ rays and neutrons have revealed dose and dose-rate dependence and RBE of neutrons [20]. In addition, the increasing use of radiation in medical diagnostics and tumor treatment, as well as accidental exposures, this study aimed to examine the risks associated with age at exposure, from the embryonic stage to after birth. Regarding the impact of age at exposure, a lifespan analysis of B6C3F1 female mice irradiated with γ rays showed that exposure during the earliest period of postnatal life is associated with the highest risk of death from cancer and other causes [21]. This strain was also used in previous studies on radiation-induced lifespan alterations associated with low-dose-rate effects and calorie restriction effects [22,23]. Since B6C3F1 mice spontaneously develop various primary tumors, including B-cell lymphomas, liver tumors, and lung tumors, irradiation further increased the frequencies of these primary tumors and resulted in lifespan shortening [21,23,24]. We have used this mouse as a model for second tumors that develop in normal tissues of patients treated for their first cancer. This is because their parental strains have different tumor susceptibilities (i.e., the B6 mice develop lymphomas whereas the C3H mice develop liver and lung tumors). Furthermore, the lifespan data of B6C3F1 mice kept in SPF conditions without irradiation have been consistent across the relevant papers, demonstrating another advantage. The risk of lethality, developing specific tumors and lifespan shortening after exposure to carbon ions and their RBE have been studied. The RBEs for acute lethality (LD50/30) from carbon ions were reported using female C57BL/6J mice (13 keV/μm) [25] and C3H/He mice (40–50 keV/μm) [26]. The RBEs for tumor development from low LET (i.e., ~ 13 keV/μm) carbon ions were previously reported for breast cancer incidence in female Sprague-Dawley rats irradiated at various ages [27], intestinal and colon tumor frequency in male and female APC^1638N^/^+^ mice exposed at 6–8 weeks of age [28], lung cancer mortality in male and female B6C3F1 mice exposed at 7 weeks of age [29], and medulloblastoma mortality in *Ptch1* heterozygous mice exposed at day 1, 4 and 10 days after birth [30], revealing the effects of age and gender. Few studies have addressed the mortality risk and its RBE after exposing animals of various ages, including young ones, to low-LET carbon-ion radiation. This would constitute important basic data for estimating the risk of developing late-occurring complications, including cancers and non-cancer complications, due to exposure to carbon ions as space radiation as well as radiotherapy.

Here, we analyzed the lifespans of male and female B6C3F1 mice exposed to various doses of γ rays or 13 keV/µm carbon ions at different ages to determine the influence of sex and age and to assess whether these factors affected the RBE for lifespan shortening.

## Materials and methods

### Mice

Male and female F1 hybrid mice were produced by crossing female C57BL/6NCrlCrlj with male C3H/HeNCrlCrlj mice purchased from Jackson Laboratory Japan, Inc., (Yokohama, Japan). They were brought into a specific pathogen-free (SPF) animal facility at the National Institutes for Quantum Science and Technology (QST; Chiba, Japan). Routine microbiological screening of control mice confirmed maintenance of the SPF condition. Three to four days after birth, the sex of pups was determined by visual inspection, and they were blindly reassigned to the nursing dams to control the size (six per dam) and sex ratio (three males and three females per dam) of the litters. All pups in each new litter were assigned to an identical treatment group. After weaning at age 28 days, males and females were housed separately (five per cage), and the cages were changed weekly. Mice were provided with autoclaved wood-shaving bedding and fed a radiosterilized diet (30 kGy; MBR-1, Funabashi Farm Co., Funabashi, Japan) and water ad libitum (water changed twice weekly). The facility was maintained at 23 ± 3°C with a relative humidity of 50 ± 10% and a 12-h light-dark cycle.

Mice were inspected daily for the remainder of their lifetime by experienced researchers, and when they were judged to reach one of pre-determined humane endpoints including abnormal posture, abnormal respiratory movement, anemia, and large tumor burden, they were euthanized by exsanguination under isoflurane anesthesia (~4% in air) within 2 hours and necropsied. Whereas most of the historical lifespan studies [21,22,31,32] used death as endpoints, the present study and our recent study [23] employed humane endpoints following recent guidelines for animal use. In the last two studies, fewer than 10% of mice died before reaching the humane endpoint. The total number of mice used in these experiments was 463, 3343, and 2634 for the control, γ rays, and carbon ions irradiated groups, respectively; the exact number of mice in each irradiated group is shown in Results. The data and tissue samples from this experiment were stored in the Japan StoreHouse of Animal Radiobiology Experiments (J-SHARE) system [33]. All experimental procedures in this study were conducted according to the protocol approved by the Institutional Animal Care and Use Committee of QST (approval numbers 07–1017 and 10–2019). The welfare of animals at the institution was reviewed by an external program of the Japanese Association of Laboratory Animal Facilities of the National University Corporations and the Japanese Association of Laboratory Animal Facilities of Public and Private Universities. All research staff have received special annual training in the care of animals. Part of the result of this series of experiments has been published elsewhere [29].

### Irradiation protocol

Mice were irradiated as outlined in Table 1. Pregnant C57BL/6NCrlCrlj mice mated with C3H/HeNCrlCrlj males (postcoital days 3, 13, and 17) and postnatal B6C3F1 mice (1, 3, 7, and 15 weeks after birth) were singly whole-body irradiated with γ rays or carbon ions at 0.2, 0.5, 1.0, 2.0, 3.0, 4.0, or 4.8 Gy without anesthesia during irradiation. The remaining mice were not irradiated as controls. γ-Irradiation was performed using a ^137^Cs source (Gammacell-40, Nordion, Ottawa, Ontario, Canada) at a dose rate of ~0.5 Gy/min. This exposure was performed inside the SPF animal facility using a ventilated acrylate polymer container (panel A in S1 Fig). For carbon-ion irradiation, the dosimetry was performed every experimental day before irradiation. The mice were transported to the synchrotron building (~200 m distance from the SPF facility), irradiated, and transported back to the SPF facility in air-tight acrylate polymer containers specially designed for this experiment (Sanki Industry Co., Ltd., Chiba, Japan) (each supplied with air sterilized by a 0.22-μm–pore polyvinylidene fluoride filter) (panel B and C in S1 Fig). The number of mice in a container depended on their age—two for pregnant dams, two for 15-week-old mice, three for 7-week-old mice, six for 3-week-old mice, and six to seven for 1-week-old mice—so that the volume occupied by the mice were almost equal. The mice were exposed to a single dose of 0 to 4.8 Gy of monoenergetic carbon ions (290 MeV/u) at nominal dose rates of 4.0 Gy/min (for 4.0 and 4.8 Gy), 1.0 Gy/min (for 1.0 and 1.2 Gy), 0.5 Gy/min (for 0.5 Gy), or 0.1 Gy/min (for 0.2 Gy) with mesh attenuators to control the particle fluence without significantly altering the beam characteristics. Previous studies have indicated that the dose rate does not change cell killing and carcinogenesis in the range of 0.1–4 Gy/min) [34–36]. The irradiation duration for the mice was between 1 and 2 min. Each mouse was subjected to whole-body irradiation with carbon ions with an estimated dose-averaged LET of ~13 keV/μm, considering the body size (less than 30 mm) and thickness of the inner container (2 mmH_2_O) and the polymethyl methacrylate (PMMA) devices (23.2 mmH_2_O), which were in total 55.2 mmH_2_O, placed upstream of the beam. The carbon ion beam was spread to ø100 mm, and the depth-dose of distribution of the monoenergetic carbon ion beam of 290 MeV/u is flat by at least 100 mmH_2_O, as reported by Kanai et al. [37]. The work was conducted as part of the joint use of the Research Project with Heavy Ions at QST (15J292).

**Table 1:** Experimental groups.

Symbol	Age at irradiation	Stage of development	Dose (Gy)
0 Gy	Not irradiated	Not applicable	0
E3	Embryonic day 3	Fetal, pre-implantation	0.2, 1.0^a^
E13	Embryonic day 13	Fetal, major organogenesis	0.2, 1.0^a^
E17	Embryonic day 17	Fetal, late stage	0.2, 0.5, 1.0, 2.0, 4.0^a^
1W	1 week after birth	Neonatal	0.2, 0.5, 1.0, 2.0, 3.0, 4.0, 4.8
3W	3 weeks after birth	Prepubertal	0.2, 0.5, 1.0, 2.0, 4.0
7W	7 weeks after birth	Early adulthood	0.2, 0.5, 1.0, 2.0, 3.0, 4.0, 4.8
15W	15 weeks after birth	Full adulthood	0.2, 1.0^a^, 2.0

Male and female mice were irradiated with γ rays or carbon ions.

^a^Only γ-ray irradiation.

### Statistics

Statistical analyses were performed using SPSS Statistics v.28 (IBM, Armonk, NY, USA). The survival rate of each group was visualized by Kaplan-Meier survival curves, and the statistical significance of differences in survival between groups was determined by the log-rank test. Cox’s proportional hazard analysis of survival rate was performed to calculate the hazard ratio for each group. Differences were considered significant at *P* < 0.05. The relationship between dose and hazard ratio point estimate was fitted to a linear equation with a *y*-intercept of 1 by the least-squares method using Excel (Microsoft, Redmond, WA, USA). Following the general definition of RBE as the ratio of dose of the two radiation types that result in an identical biological effect, RBE was calculated as the ratio of the slopes of the dose-response of the two radiation types. The standard error (SE) for each ratio value was calculated using the delta method.

## Results

### Lifespan shortening

To determine any correlation between age at exposure, sex, or radiation type and the lifespan of mice irradiated with γ rays or carbon ions, male and female B6C3F1 mice at various developmental stages, namely embryonic day 3 (E3), E13, and E17 and postnatal weeks 1, 3, 7, and 15, were irradiated with ^137^Cs γ rays or 290 MeV/u monoenergetic carbon ions (LET ~ 13 keV/µm) at doses indicated in Table 1. To enable comparison, a group of non-irradiated mice (control) was analyzed along with the irradiated mice.

Table 2 shows the number of mice and the average lifespan in each irradiation group by age at exposure, sex, and doses. The average lifespan of non-irradiated mice was 862 days (males) and 872 days (females). This lifespan aligns with previous data for B6C3F1 female mice, such as 871.1 days and 860.5 days reported by Sasaki [21] and Tanaka [22], respectively. For example, in male mice irradiated with γ rays at age 1 week, the average lifespan after irradiation with 0.2 Gy, 2.0 Gy, or 4.0 Gy was 825 days (reduction ratio: –4.4%), 710 days (–18%), and 522 days (–40%), respectively; for females, the corresponding values were 833 days (–4.5%), 703 days (–19%), and 489 days (–44%) (Table 2). For both sexes, lifespan shortening was dependent on radiation dose. For male mice irradiated with carbon ions at age 1 week, the average lifespan after irradiation with 0.2 Gy, 2 Gy, or 4 Gy was 799 days (–7.3%), 665 days (–23%), and 419 days (–51%), respectively; for females, the corresponding values were 792 days (–9%), 645 days (–26%), and 409 days (–53%) (Table 2). Like γ-irradiation, carbon-ion irradiation resulted in a dose-dependent reduction in lifespan, with carbon ions having a slightly greater and more significant effect on females.

**Table 2:** Lifespan and hazard ratio values after irradiation of male and female mice at various ages with γ rays and carbon ions.

Carbon ions	Male					Female				
Age	Dose (Gy)	*N* ^a^	Mean lifespan ± SE (days)	*P* value	Hazard ratio (95% CI^b^)	Dose (Gy)	*N* ^a^	Mean lifespan ± SE (days)	*P* value	Hazard ratio (95% CI^b^)
Control	0	228	862.4 ± 11.5	0	1	0	235	871.9 ± 10.2	0	1
E3	0.2	56	879.1 ± 27.6	0.246	0.84 (0.63, 1.13)	0.2	58	830.2 ± 19.2	0.051	1.33 (1.00, 1.78)
	1.0	47	888.1 ± 23.9	0.467	0.89 (0.65, 1.22)	1.0	50	900.6 ± 20.5	0.345	0.86 (0.63, 1.17)
E13	0.2	51	838.5 ± 19.0	0.06	1.34 (0.99, 1.83)	0.2	57	862.5 ± 19.8	0.691	1.06 (0.79, 1.42)
	1.0	51	875.7 ± 21.7	0.947	0.99 (0.73, 1.34)	1.0	50	807.2 ± 26.4	0.086	1.31 (0.96, 1.78)
E17	0.2	55	871.2 ± 23.1	0.769	0.96 (0.71, 1.29)	0.2	55	851.0 ± 23.8	0.746	1.05 (0.78, 1.41)
	0.5	51	863.9 ± 28.2	0.677	0.94 (0.69, 1.27)	0.5	49	896.2 ± 17.8	0.795	0.96 (0.71, 1.31)
	1.0	49	810.2 ± 27.2	0.27	1.19 (0.87, 1.62)	1.0	50	853.3 ± 25.5	0.651	1.07 (0.79, 1.46)
	2.0	56	795.5 ± 30.4	0.169	1.23 (0.92, 1.65)	2.0	52	775.0 ± 24.5	0.002**	1.61 (1.19, 2.18)
	4.0	44	402.1 ± 51.0	< 0.001***	4.23 (3.05, 5.88)	4.0	48	471.9 ± 47.4	< 0.001***	5.16 (3.74, 7.14)
1W	0.2	52	824.5 ± 22.6	0.167	1.24 (0.92, 1.67)	0.2	49	833.1 ± 24.2	0.168	1.24 (0.91, 1.69)
	0.5	50	779.1 ± 28.8	0.021*	1.44 (1.06, 1.96)	0.5	50	789.8 ± 22.6	0.001**	1.71 (1.25, 2.33)
	1.0	71	796.9 ± 24.5	0.082	1.27 (0.97, 1.66)	1.0	70	773.0 ± 19.7	< 0.001***	1.74 (1.33, 2.28)
	2.0	50	710.3 ± 25.2	< 0.001***	2.13 (1.56, 2.90)	2.0	52	702.9 ± 22.5	< 0.001***	3.11 (2.28, 4.24)
	3.0	50	649.0 ± 25.7	< 0.001***	3.19 (2.33, 4.37)	3.0	50	589.0 ± 26.4	< 0.001***	5.98 (4.32, 8.27)
	4.0	82	521.5 ± 29.7	< 0.001***	3.80 (2.93, 4.92)	4.0	82	489.0 ± 25.7	< 0.001***	8.28 (6.26, 10.95)
	4.8	50	444.8 ± 31.1	< 0.001***	7.47 (5.41, 10.31)	4.8	50	484.3 ± 31.2	< 0.001***	8.78 (6.33, 12 20)
3W	0.2	51	853.3 ± 25.9	0.682	0.94 (0.69, 1.28)	0.2	51	853.6 ± 21.2	0.474	1.12 (0.83, 1.51)
	0.5	50	849.6 ± 21.8	0.351	1.16 (0.85, 1.58)	0.5	48	834.4 ± 25.8	0.523	1.11 (0.81, 1.51)
	1.0	49	807.7 ± 27.2	0.069	1.33 (0.98, 1.82)	1.0	46	768.5 ± 24.8	< 0.001***	1.92 (1.39, 2.64)
	2.0	51	740.4 ± 22.4	< 0.001***	2.17 (1.59, 2.95)	2.0	51	675.9 ± 22.8	< 0.001***	3.72 (2.71, 5.12)
	4.0	82	631.8 ± 24.8	< 0.001***	3.09 (2.39, 4.01)	4.0	80	530.2 ± 26.1	< 0.001***	6.15 (4.67, 8.10)
7W	0.2	51	851.1 ± 22.0	0.358	1.15 (0.85, 1.56)	0.2	51	831.5 ± 25.1	0.252	1.19 (0.88, 1.62)
	0.5	50	815.8 ± 22.3	0.038*	1.39 (1.02, 1.89)	0.5	50	836.1 ± 24.3	0.351	1.16 (0.85, 1.57)
	1.0	70	805.3 ± 21.1	0.02*	1.38 (1.05, 1.80)	1.0	70	777.7 ± 21.8	< 0.001***	1.70 (1.30, 2.22)
	2.0	51	807.0 ± 18.4	0.002**	1.62 (1.19, 2.20)	2.0	51	706.6 ± 26.0	< 0.001***	2.68 (1.96, 3.65)
	3.0	50	684.9 ± 31.0	< 0.001***	2.64 (1.93, 3.61)	3.0	50	657.5 ± 24.9	< 0.001***	3.96 (2.88, 5.44)
	4.0	50	635.1 ± 25.0	< 0.001***	4.27 (3.11, 5.88)	4.0	50	620.3 ± 29.1	< 0.001***	4.57 (3.32, 6.31)
	4.8	50	668.8 ± 27.4	< 0.001***	3.31 (2.41, 4.54)	4.8	50	596.5 ± 25.6	< 0.001***	5.56 (4.03, 7.67)
15W	0.2	51	806.2 ± 25.2	0.021*	1.43 (1.06, 1.95)	0.2	51	831.0 ± 20.8	0.096	1.29 (0.96, 1.75)
	1.0	50	818.4 ± 24.6	0.117	1.28 (0.94, 1.74)	1.0	49	770.3 ± 23.2	< 0.001***	2.02 (1.47, 2.76)
	2.0	51	785.9 ± 25.8	0.017*	1.45 (1.07, 1.97)	2.0	51	726.4 ± 21.9	< 0.001***	2.83 (2.07, 3.87)
**Carbon ions**	**Male**					**Female**				
**Age**	**Dose (Gy)**	***n*** ^a^	**Mean lifespan ± SE (days)**	***P* value**	**Hazard ratio** **(95% CI**^b^)	**Dose (Gy)**	***n*** ^a^	**Mean lifespan ± SE (days)**	***P* value**	**Hazard ratio** **(95% CI**^b^)
Control	0	228	862.4 ± 11.5	0	1	0	235	871.9 ± 10.2	0	1
E3	0.2	71	846.1 ± 23.2	0.961	1.01 (0.77, 1.32)	0.2	73	859.6 ± 18.9	0.678	1.06 (0.81, 1.38)
E13	0.2	48	775.9 ± 34.0	0.087	1.31 (0.96, 1.80)	0.2	42	857.7 ± 24.6	0.569	1.10 (0.79, 1.53)
E17	0.2	45	811.3 ± 33.7	0.349	1.17 (0.85, 1.61)	0.2	45	806.8 ± 26.3	0.043*	1.39 (1.01, 1.92)
	0.5	40	760.2 ± 40.3	0.161	1.27 (0.91, 1.78)	0.5	40	855.7 ± 25.2	0.589	1.20 (0.78, 1.54)
	1.0	36	790.0 ± 44.9	0.759	1.06 (0.74, 1.51)	1.0	40	806.6 ± 24.2	0.03*	1.45 (1.04, 2.04)
	2.0	41	544.2 ± 38.0	< 0.001***	4.10 (2.90, 5.79)	2.0	45	709.0 ± 39.3	< 0.001***	1.86 (1.34, 2.56)
1W	0.2	45	799.2 ± 28.4	0.065	1.35 (0.98, 1.87)	0.2	49	792.2 ± 20.3	< 0.001***	1.79 (1.31, 2.45)
	0.5	80	804.0 ± 19.8	0.013*	1.39 (1.07, 1.79)	0.5	80	820.6 ± 17.4	0.01*	1.40 (1.09, 1.81)
	1.0	93	774.1 ± 18.2	< 0.001***	1.67 (1.31, 2.13)	1.0	97	765.2 ± 13.9	< 0.001***	2.21 (1.73, 2.82)
	2.0	81	665.2 ± 20.1	< 0.001***	3.38 (2.59, 4.32)	2.0	81	644.9 ± 19.7	< 0.001***	4.42 (3.37, 5.79)
	3.0	41	585.3 ± 29.3	< 0.001***	5.74 (4.05, 8.17)	3.0	41	548.2 ± 31.1	< 0.001***	7.19 (5.07, 10.21)
	4.0	41	418.9 ± 33.1	< 0.001***	12.53 (8.71, 18.03)	4.0	41	409.0 ± 36.0	< 0.001***	12.36 (8.65, 17.67)
	4.8	47	323.0 ± 34.8	< 0.001***	15.02 (10.68, 21.12)	4.8	41	279.3 ± 30.4	< 0.001***	27.58 (19.01, 40.0)
3W	0.2	48	861.1 ± 24.8	0.912	1.02 (0.75, 1.39)	0.2	52	859.8 ± 19.8	0.53	1.10 (0.82, 1.49)
	0.5	40	790.5 ± 32.4	0.127	1.30 (0.93, 1.82)	0.5	39	786.6 ± 25.2	0.006**	1.61 (1.15, 2.27)
	1.0	38	758.7 ± 23.7	< 0.001***	1.96 (1.39, 2.78)	1.0	40	763.5 ± 30.2	0.003**	1.66 (1.19, 2.33)
	2.0	79	725.1 ± 20.1	< 0.001***	2.08 (1.60, 2.70)	2.0	85	687.9 ± 19.5	< 0.001***	3.32 (2.55, 4.32)
	4.0	36	596.8 ± 34.0	< 0.001***	4.46 (3.10, 6.41)	4.0	40	509.7 ± 36.4	< 0.001***	7.45 (5.21, 10.64)
7W	0.2	54	793.3 ± 21.9	0.003**	1.58 (1.17, 2.14)	0.2	47	834.1 ± 21.5	0.052	1.37 (1.00, 1.87)
	0.5	40	855.5 ± 24.7	0.65	1.08 (0.77, 1.52)	0.5	41	754.7 ± 21.7	< 0.001***	2.50 (1.77, 3.51)
	1.0	60	782.1 ± 23.4	0.003**	1.55 (1.17, 2.07)	1.0	61	771.2 ± 19.0	< 0.001***	2.08 (1.56, 2.77)
	2.0	41	768.3 ± 24.0	< 0.001***	1.95 (1.39, 2.73)	2.0	48	686.6 ± 23.4	< 0.001***	3.45(2.50, 4.77)
	4.0	40	605.1 ± 35.6	< 0.001***	3.71 (2.63, 5.25)	4.0	40	526.1 ± 32.1	< 0.001***	7.80 (5.47, 11.13)
	4.8	40	647.4 ± 29.4	< 0.001***	3.81 (2.68, 5.42)	4.8	42	449.3 ± 28.9	< 0.001***	14.00 (9.71, 20.19)
15W	0.2	40	819.6 ± 32.8	0.38	1.16 (0.83, 1.63)	0.2	40	806.1 ± 20.0	0.002**	1.71 (1.22, 2.40)
	2.0	40	774.3 ± 26.6	0.002**	1.69 (1.21, 2.38)	2.0	39	694.0 ± 24.9	< 0.001***	3.70 (2.59, 5.28)

^a^Number of animals. ^b^CI, confidence interval. *P < 0.05, **P < 0.01, ***P < 0.001 vs. control.

Regarding the influence of age at exposure, the effects of 2 Gy irradiation at various ages (E17 and 1, 3, 7, and 15 weeks after birth) were as follows. For male mice irradiated with γ rays (Table 2), the respective average lifespan was 796 days (–8%), 710 days (–18%) (mentioned above), 740 days (–14%), 807 days (–6%), and 786 days (–9%); for females, the corresponding values were 775 days (–11%), 703 days (–19%) (mentioned above), 676 days (–22%), 707 days (–19%), and 726 days (–17%). For male mice irradiated with carbon ions (Table 2), the respective average lifespan values were 544 days (–37%), 665 days (–23%) (mentioned above), 725 days (–16%), 768 days (–11%), and 774 days (–10%); for females, the corresponding values were 709 days (–19%), 645 days (–26%) (mentioned above), 688 days (–21%), 687 days (–21%), and 694 days (–20%). As detailed below, the death of males irradiated at E17 included early deaths most likely related to acute or subacute effects. Thus, the effect on lifespan shortening was greatest for mice irradiated at age 1 week, for irradiation with carbon ions (versus γ rays).

### Survival rate

**Fig 1:**
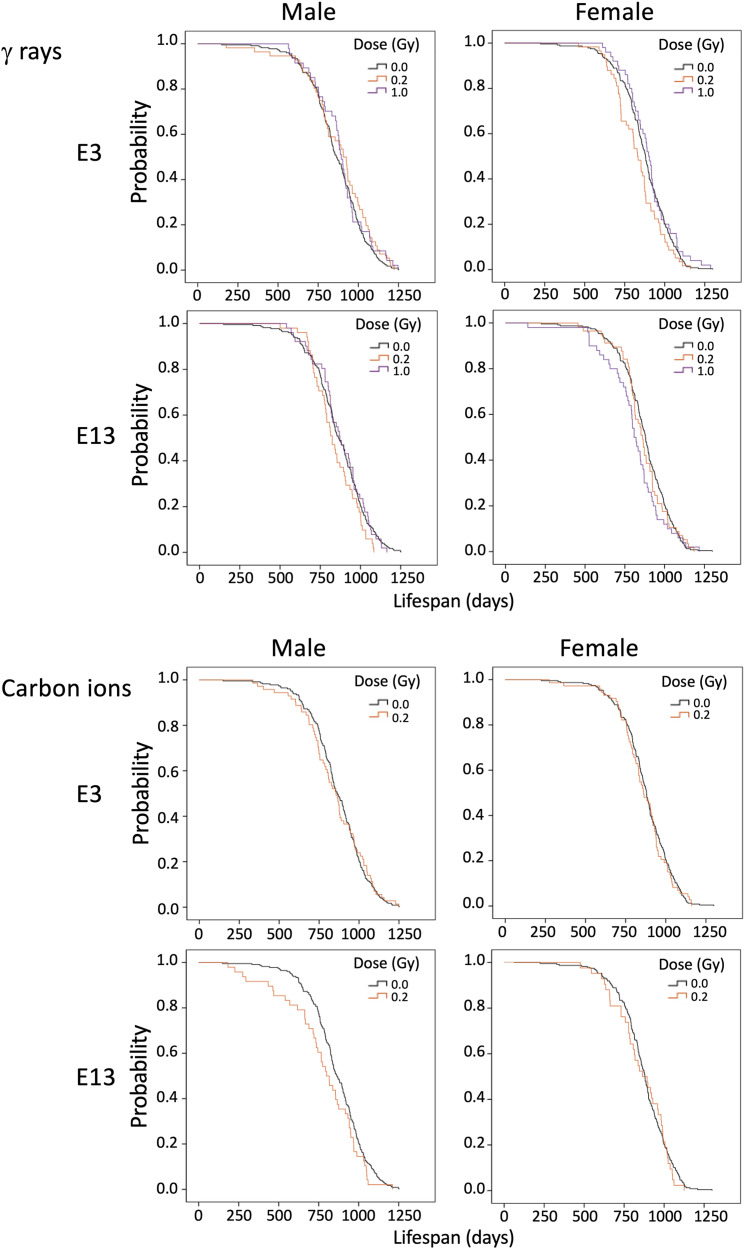
Kaplan-Meier survival curves for mice irradiated with various doses at E3 and E13 (γ rays and carbon ions). Data for non-irradiated control mice are presented alongside data for irradiated mice, with males and females displayed side by side. Ages at irradiation were embryonic day 3 (E3) and E13 to γ rays (upper panels) or carbon ions (lower panels). *P < 0.05; **P < 0.01; ***P < 0.001 versus 0 Gy by the log-rank test.

**Fig 2:**
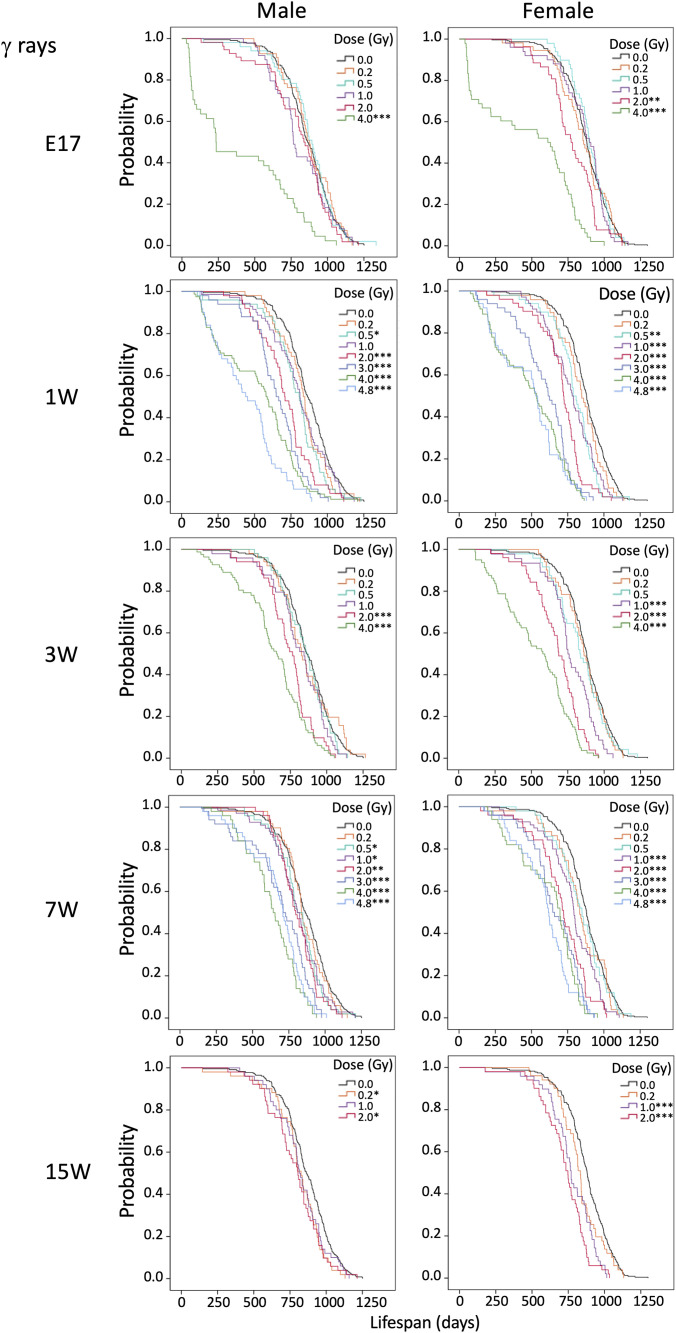
Kaplan-Meier survival curves for mice irradiated with various doses at various ages (γ rays). Data for non-irradiated control mice are presented alongside data for irradiated mice, with males and females displayed side by side. Ages at irradiation were embryonic day 17 (E17) and postnatal weeks 1 (1W), 3W, 7W, and 15W to γ rays. *P < 0.05; **P < 0.01; ***P < 0.001 versus 0 Gy by the log-rank test.

**Fig 3:**
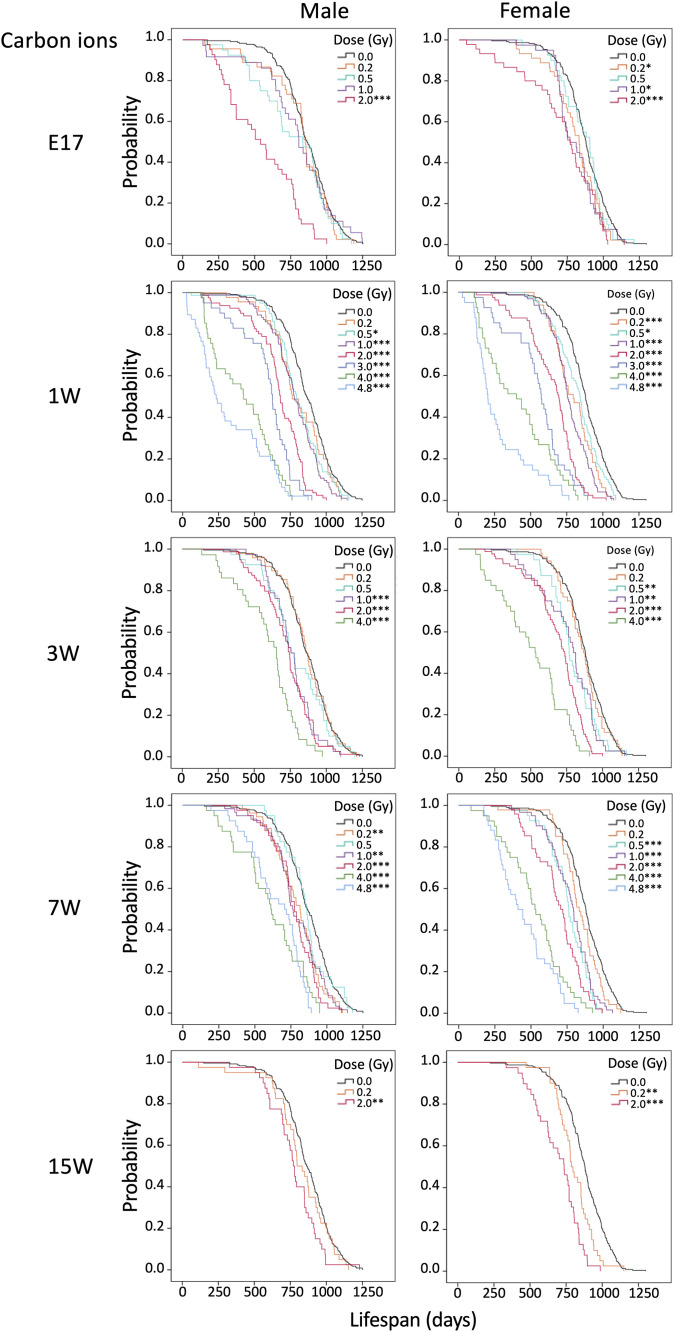
Kaplan-Meier survival curves for mice irradiated with various doses at various ages (carbon ions). Data for non-irradiated control mice are presented alongside data for irradiated mice, with males and females displayed side by side. Ages at irradiation were embryonic day 17 (E17) and postnatal weeks 1 (1W), 3W, 7W, and 15W to carbon ions. *P < 0.05; **P < 0.01; ***P < 0.001 versus 0 Gy by the log-rank test.

Next, we assessed the survival of irradiated mice. Figs 1–3 displays Kaplan-Meier curves for the survival rates of both males (left panels) and females (right panels) following irradiation with various doses of γ rays or carbon ions at various ages. The data for E3 and E13 irradiated with γ rays (upper panels) or carbon ions (lower panels) are shown in Fig 1. The data for E17, 1W, 3W, 7W, and 15W irradiated with γ rays are shown in Fig 2, and the data for the same samples irradiated with carbon ions are shown in Fig 3. The groups irradiated at each dose that showed significant differences analyzed by the log-rank test compared to the 0 Gy group are marked with asterisks in Figs 1–3.

For irradiation at E3 or E13, note that only the lower doses (0.2–1.0 Gy) were used, as we found that irradiation with 2.0 Gy at these ages (i.e., pre-implantation and during major organogenesis) resulted in acute effects such as miscarriage, hydrocephalus, and limb malformation. No significant lifespan shortening was observed for irradiation at E3 or E13 with γ rays (0.2 or 1.0 Gy) (Fig 1 upper) or carbon ions (0.2 Gy) (Fig 1 lower). γ-Irradiation at E17 significantly shortened the lifespan of male mice at the dose of 4.0 Gy but not 2.0 Gy or less, and likewise for female mice at a dose of 2.0 or 4.0 Gy but not 1.0 Gy or less (Fig 2). Carbon-ion irradiation at E17 reduced the lifespan of male mice significantly at 2.0 Gy but not 1.0 Gy or less, and that of female mice significantly at 0.2 Gy, 1.0 Gy, and 2.0 Gy but not 0.5 Gy (Fig 3). γ-Irradiation at E17 with 4.0 Gy induced death due to early effects in males and females, as did 2.0 Gy of carbon ions for males, which led us to exclude these data from the subsequent risk analysis.

Regarding postnatal mice, lifespan was significantly reduced for males irradiated with γ rays (Fig 2) at a dose of 0.5 Gy or 2.0 Gy or more at 1 week of age, 2.0 Gy or more at age 3 weeks, 0.5 Gy or more at age 7 weeks, and 0.2 Gy or 2.0 Gy at age 15 weeks. For γ-irradiated females, significant lifespan reduction was observed at a dose of 0.5 Gy or more at age 1 week and 1.0 Gy or more at age 3, 7, or 15 weeks. For carbon ions (Fig 3), lifespan was significantly reduced for male mice irradiated with 0.5 Gy or more at age 1 week, 1.0 Gy or more at age 3 weeks, 0.2 and 1.0 Gy or more at age 7 weeks, and 2.0 Gy at age 15 weeks. For females, significant reduction was observed with 0.2 Gy or more at age 1 week, 0.5 Gy or more at age 3 or 7 weeks, and 0.2 Gy or more at age 15 weeks.

Exposure at E17 had minimal effects on lifespan for both males and females, in contrast to exposure after birth. Lifespan was shortened in a dose-dependent manner for all experimental groups and was more substantial with carbon ions than γ rays.

### Hazard ratio for mortality

**Fig 4:**
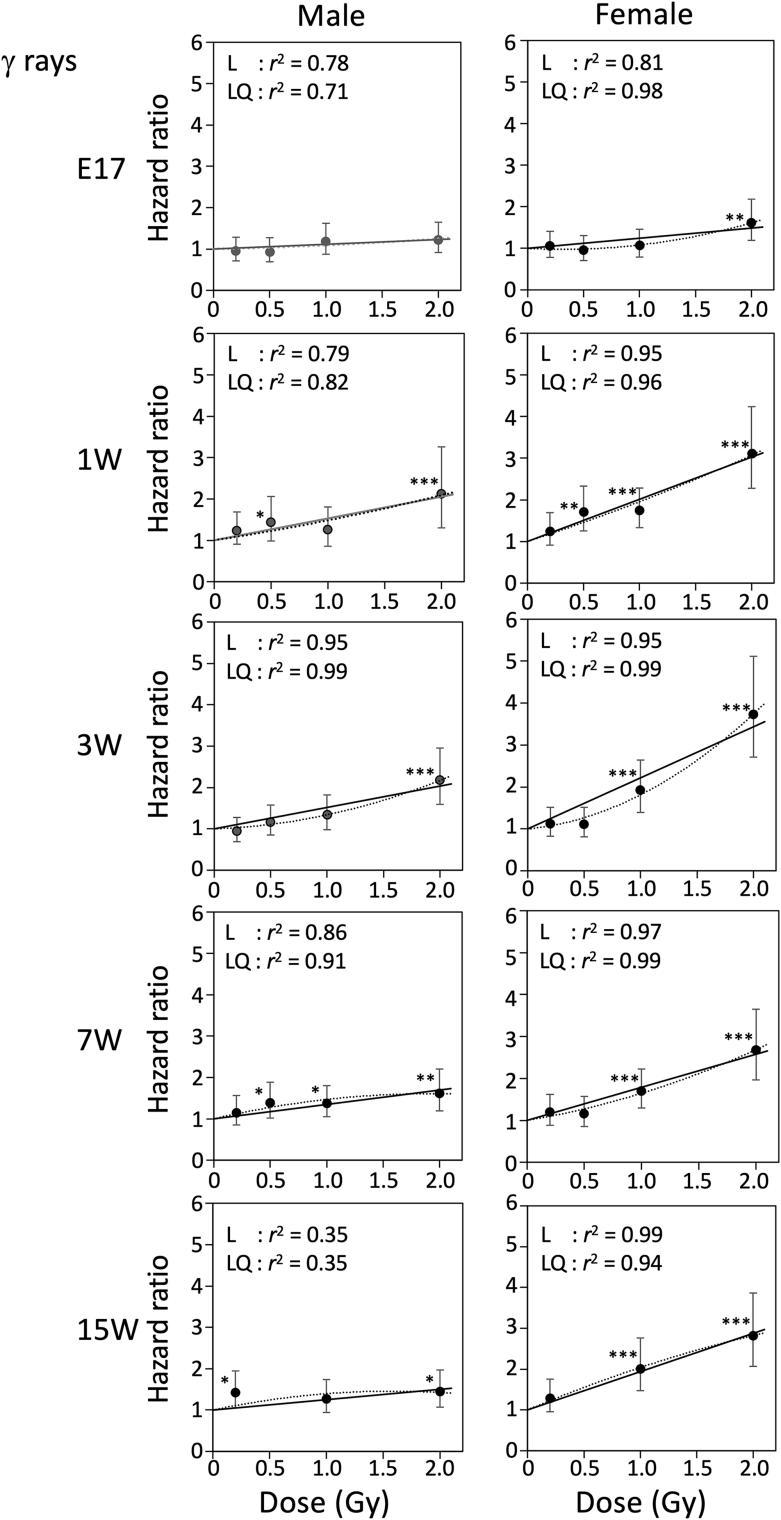
Relationships between hazard ratio and radiation dose (γ rays, 0-2 Gy). Relationship between the hazard ratio and radiation dose of γ rays over the dose range of 0–2 Gy. Ages at irradiation were embryonic day 17 (E17) and postnatal week 1 (1W), 3W, 7W and 15W. Vertical bars indicate the 95% confidence interval. Linear fitting or linear-quadratic fitting was performed by the least-squares method, and *r*^2^ values for each dose range are indicated. *P < 0.05; **P < 0.01; ***P < 0.001 versus 0 Gy.

**Fig 5:**
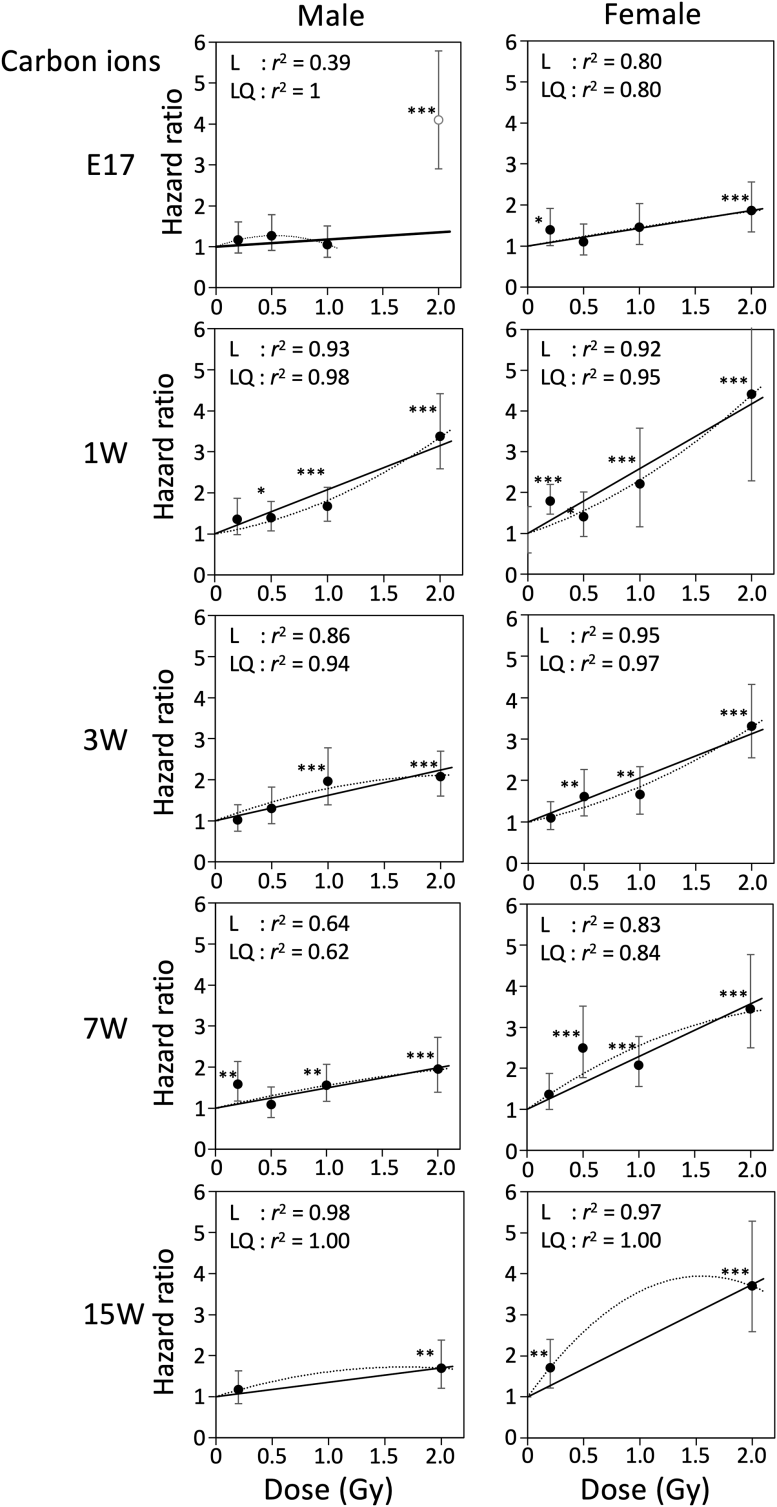
Relationships between hazard ratio and radiation dose (carbon ions, 0-2 Gy). Relationship between the hazard ratio and radiation dose of carbon ions over the dose range of 0–2 Gy. Ages at irradiation were embryonic day 17 (E17) and postnatal week 1 (1W), 3W, 7W and 15W. Vertical bars indicate the 95% confidence interval. Linear fitting or linear-quadratic fitting was performed by the least-squares method, and *r*^2^ values for each dose range are indicated. *P < 0.05; **P < 0.01; ***P < 0.001 versus 0 Gy.

**Fig 6:**
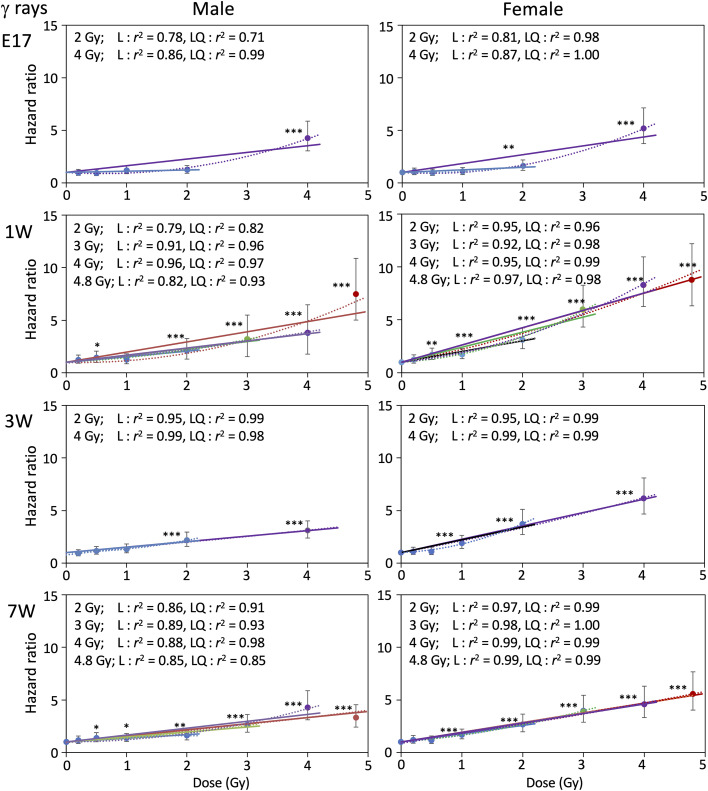
Relationships between hazard ratio and radiation dose (γ rays, 0-4.8 Gy). The relationship between the hazard ratio and radiation dose of γ rays over the dose range of 0–4.8 Gy. Ages at irradiation were embryonic day 17 (E17) and postnatal week 1 (1W), 3W and 7W. Vertical bars indicate the 95% confidence interval. Linear fitting or linear-quadratic fitting was performed by the least-squares method, and *r*^2^ values for each dose range are indicated. *P < 0.05; **P < 0.01; ***P < 0.001 versus 0 Gy.

**Fig 7:**
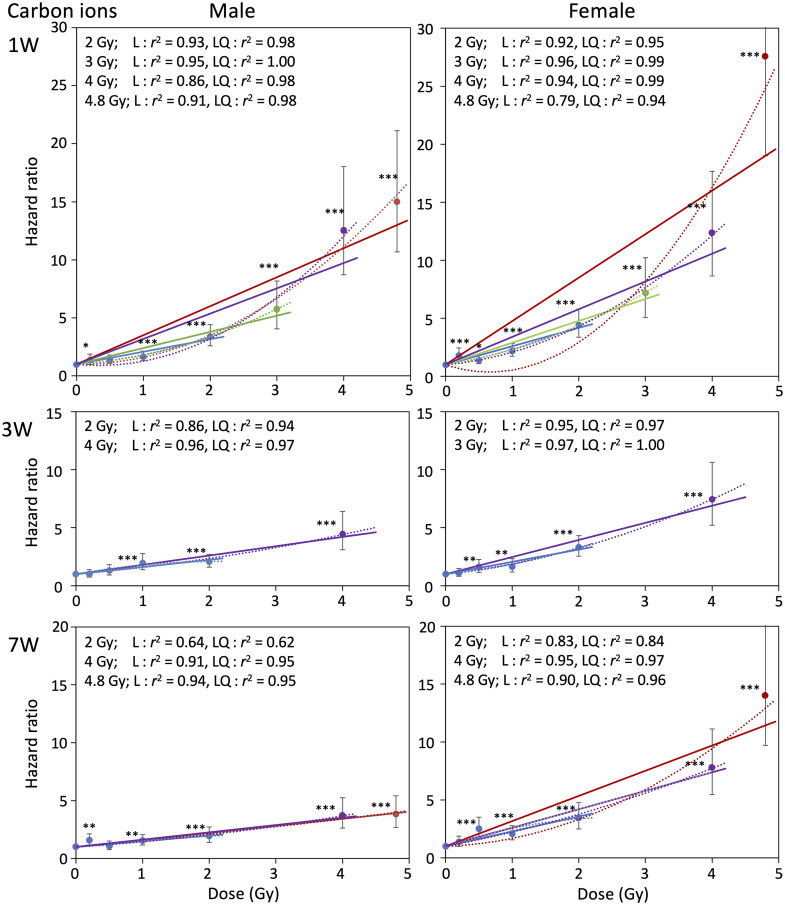
Relationships between hazard ratio and radiation dose (carbon ions, 0-4.8 Gy). The relationship between the hazard ratio and radiation dose of carbon ions over the dose range of 0–4.8 Gy. Ages at irradiation were postnatal week 1 (1W), 3W and 7W. Vertical bars indicate the 95% confidence interval. Linear fitting or linear-quadratic fitting was performed by the least-squares method, and *r*^2^ values for each dose range are indicated. *P < 0.05; **P < 0.01; ***P < 0.001 versus 0 Gy.

We next calculated the hazard ratio for mortality of male and female mice irradiated at various ages with γ rays or carbon ions, using Cox’s proportional hazard model (Table 2). Figs 4 and 5 show relationships between hazard ratio and radiation dose for γ rays and carbon ions, respectively, with male and female data displayed side by side, over the dose range 0–2 Gy. The hazard ratio increased dose-dependently for all experimental groups in the range 0–2 Gy, except for the 2 Gy point of carbon ions with E17 males (Fig 5), for which early death within 1 year after exposure increased the hazard ratio substantially. The response was adequately fitted to linear equations with a y-intercept of 1, and the fit was only slightly improved by adding quadratic terms (see *r*^2^ values in Figs 4 and 5). Results for the full dose range (0–4 Gy or 4.8 Gy) are presented in Figs 6 and 7 for γ-ray and carbon ions, respectively (for relevant groups only), where fitting to linear and linear-quadratic equations was performed. Over the full dose range, the hazard ratio after γ-ray or carbon-ion irradiation increased linearly with dose, with the exceptions of males and females irradiated at age 1 week or earlier and females irradiated with carbon ions at age 7 weeks, for which the response was more like linear quadratic. The slopes of the dose response for females were always steeper than those for males, suggesting that females tended to be more susceptible than males to either radiation type (Figs 4–7).

### Excess relative risk for lifespan shortening

**Fig 8:**
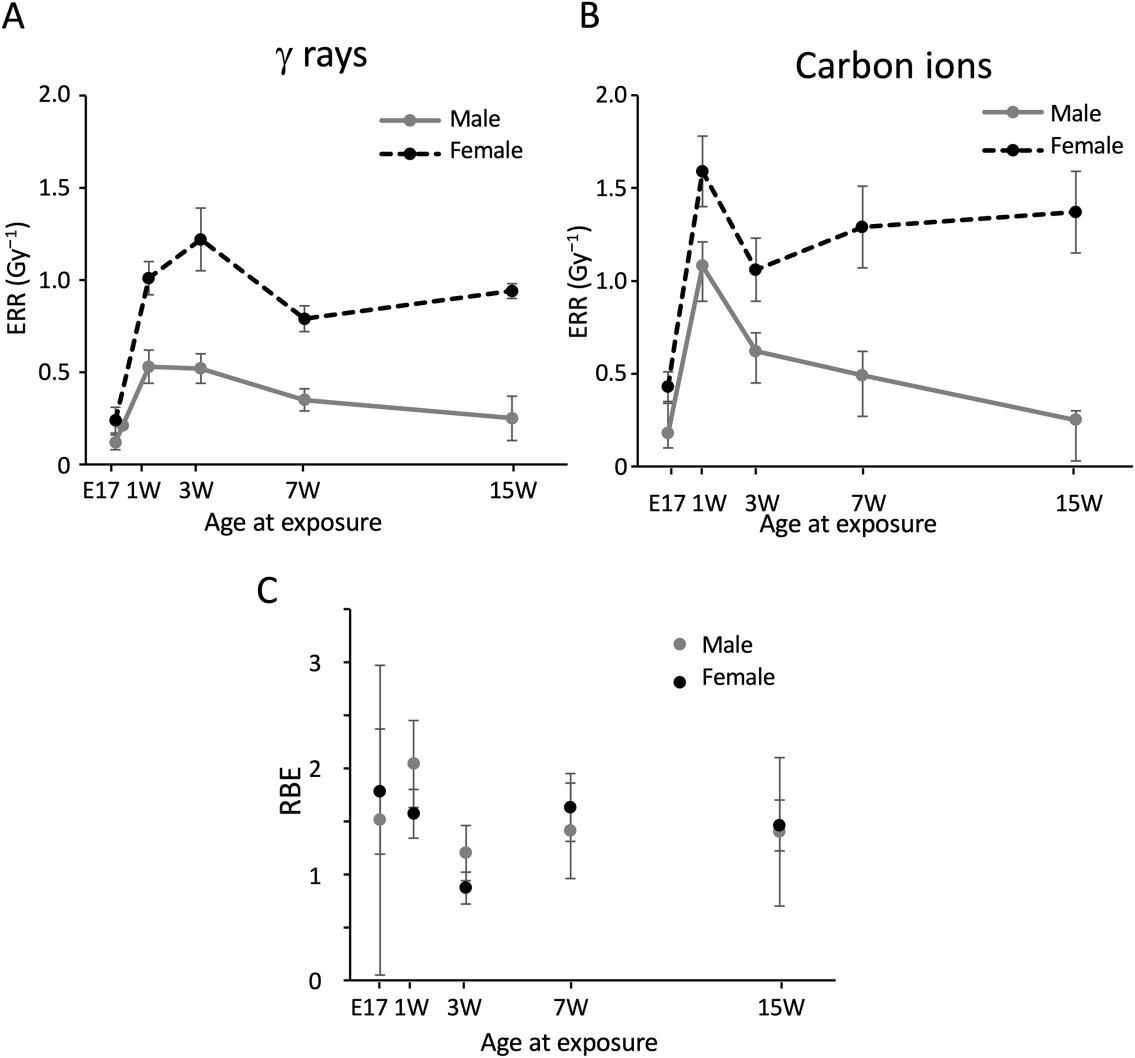
ERR for lifespan shortening and RBE of carbon ions for lifespan shortening at various ages. **(A)** ERR for lifespan shortening after γ-ray exposure. **(B)** ERR for lifespan shortening after carbon-ion exposure. **(C)** RBE of carbon ions for lifespan shortening after exposure to carbon ions. The data from postcoital days 3 and 13 were excluded from these figures because it was not possible to accurately calculate the values based on only one or two data points. Vertical bars in panels A and B indicate the 95% confidence interval. *P < 0.05; **P < 0.01; ***P < 0.001 versus 0 Gy. Vertical bars in panel C indicate the mean ± SE.

We then calculated excess relative risk (ERR) per Gy, which was defined as the increase in hazard ratio per unit dose in the linear model; this enabled the comparison of dose response among sexes, ages at exposure, and radiation types (Table 3). Although the fit to the linear-quadratic model was good over the full dose range (Figs 6 and 7), the dose-response was rather linear over the range 0–2 Gy. For this reason, and because the linear model is frequently used in epidemiology [1], we chose to use ERR/Gy. The values for the range from 0 Gy to a given dose (2.0, 4.0, or 4.8 Gy) were obtained (Table 3), and those for the range 0–2 Gy are plotted against age at exposure (Fig 8A and B).

**Table 3:** Excess relative risk (ERR) per Gy of γ rays or carbon ions and relative biological effectiveness (RBE) of carbon ions for the lifespan of B6C3F1 male and female mice.

Sex	Age at exposure	Dose^a^ (Gy)	ERR (Gy^ − 1^) ^b^		RBE^c^
			γ rays^d^	Carbon ions^d^	Carbon ions^c^^,^^d^
Male	E17	2.0	0.12 ± 0.04	0.18 ± 0.16^e^	1.53 ± 1.48^e^
	1W	2.0	0.53 ± 0.09	1.08 ± 0.13	2.04 ± 0.42
		4.0	0.68 ± 0.04	2.18 ± 0.34	3.21 ± 0.54
		4.8	0.97 ± 0.14	2.50 ± 0.28	2.58 ± 0.47
	3W	2.0	0.52 ± 0.08	0.62 ± 0.10	1.20 ± 0.26
		4.0	0.53 ± 0.03	0.80 ± 0.61	1.54 ± 0.16
	7W	2.0	0.35 ± 0.06	0.49 ± 0.13	1.41 ± 0.45
		4.0	0.66 ± 0.09	0.63 ± 0.07	0.96 ± 0.16
		4.8	0.58 ± 0.07	0.61 ± 0.04	1.05 ± 0.15
	15W	2.0	0.25 ± 0.12	0.25 ± 0.05	1.40 ± 0.70
Female	E17	2.0	0.24 ± 0.07	0.43 ± 0.08	1.78 ± 0.59
	1W	2.0	1.01 ± 0.09	1.59 ± 0.19	1.57 ± 0.23
		4.0	1.63 ± 0.13	2.39 ± 0.05	1.47 ± 0.19
		4.8	1.63 ± 0.09	3.75 ± 0.66	2.30 ± 0.42
	3W	2.0	1.22 ± 0.17	1.06 ± 0.17	0.87 ± 0.15
		4.0	1.27 ± 0.07	1.48 ± 0.13	1.16 ± 0.12
	7W	2.0	0.79 ± 0.07	1.29 ± 0.22	1.63 ± 0.32
		4.0	0.90 ± 0.04	1.60 ± 0.13	1.77 ± 0.16
		4.8	0.92 ± 0.03	2.18 ± 0.26	2.36 ± 0.29
	15W	2.0	0.94 ± 0.04	1.37 ± 0.22	1.46 ± 0.24

^a^Maximum dose for the dose range used for ERR calculation.

^b^Defined by least-squares fitting of hazard ratio data to 1 + ERR × dose (Gy).

^c^Calculated as the ratio of ERR for carbon ions to ERR for γ rays for each age.

^d^Data represent the mean ± SE.

^e^ERR/Gy and RBE for E17 male mice were calculated using data from 0 to 2 Gy for γ rays, and from 0 to 1 Gy for carbon ions.

For γ-irradiated male mice, the largest ERR/Gy values were observed at 1 and 3 weeks of age (0.53 ± 0.09 and 0.52 ± 0.08, respectively), which decreased with age (Fig 8A). For females, ERR/Gy values were largest at ages 1 and 3 weeks (1.01 ± 0.09 and 1.22 ± 0.17, respectively) and decreased at ages 7 and 15 weeks (0.79 ± 0.07 and 0.94 ± 0.04, respectively). For male mice irradiated with carbon ions, ERR/Gy was largest at age 1 week (1.08 ± 0.13), declined sharply at age 3 weeks (0.62 ± 0.10), and further decreased at older ages (Fig 8B). For females, ERR/Gy was largest at age 1 week (1.59 ± 0.19) and sharply decreased at age 3 weeks (1.06 ± 0.17), with no further decrease at ages 7 and 15 weeks (1.29 ± 0.22 and 1.37 ± 0.22, respectively).

Thus, the ERR/Gy value was larger for females than males irradiated with γ rays or carbon ions at any age. The value for exposure at E17 was the lowest among all ages tested. The ERR/Gy value for carbon ions was larger than that for γ rays at any age tested.

### RBE of carbon ions for lifespan shortening

Finally, the RBE value of carbon ions for lifespan shortening was computed for each age at exposure and sexes. Because RBE is defined as the ratio of doses that produce the same level of a specific biological endpoint, and as shown above, the dose-response curves of the hazard ratio were considered linear, the RBE was calculated as the ratio of the ERR/Gy values for carbon ions and γ rays (Table 3). Over the dose range 0–2 Gy, the RBE values for male mice exposed at E17 or at 1, 3, 7, or 15 weeks of age were 1.53 ± 3.07, 2.04 ± 2.04, 1.20 ± 0.26, 1.41 ± 0.45, and 1.40 ± 0.70, respectively; the corresponding values for females were 1.78 ± 0.59, 1.57 ± 0.23, 0.87 ± 0.15, 1.63 ± 0.32, and 1.46 ± 0.24 (mean ± SE, Table 3 and Fig 8C). These results suggested that the effect of carbon ions (LET 13 keV/µm) on lifespan shortening was no greater than twice that of γ rays at any age at exposure.

Over the dose range 0–4 Gy, the RBE values for male mice exposed at 1, 3, or 7 weeks of age were 3.21 ± 0.54, 1.54 ± 0.16, and 0.96 ± 0.16, respectively; the corresponding values for females were 1.47 ± 0.19, 1.16 ± 0.12, and 1.77 ± 0.16 (Table 3). Over the dose range 0–4.8 Gy, the respective values for males exposed at age 1 or 7 weeks were 2.58 ± 0.47 and 1.05 ± 0.15, and the corresponding values for females were 2.30 ± 0.42 and 2.36 ± 0.29. The RBE was larger (~3) if the higher doses were included, especially for exposure at age 1 week, reflecting the linear-quadratic nature of the dose response.

## Discussion

The present study indicates that the risk (ERR/Gy) of lifespan shortening in mice due to irradiation with γ rays or carbon ions varied with age at exposure and sex. The greatest susceptibility was observed at 1 week of age for both sexes, with females being more susceptible than males and carbon ions being more potent than γ rays. The RBE of carbon ions for lifespan shortening ranged from 1 to 2, regardless of age at exposure or sex.

The effect of age at exposure on the risk of lifespan shortening was large for both γ-ray and carbon-ion irradiation early in life, and the risk decreased toward adulthood. On the other hand, no significant increase was observed in the late fetal period. These results agree with observations of survivors of atomic-bomb radiation (mostly γ rays) in that younger age correlated with a higher risk (ERR/Gy) of developing a solid cancer and that fetal exposure was associated with a relatively low risk of solid cancer compared with the risk after exposure during early childhood [1,38,39]. Similar results were reported in a study of female B6C3F1 mice, in which lifespan reduction was greatest when animals were exposed to γ radiation early in life and lower thereafter, with that in utero being negligible [21]. Our findings are consistent with previous data regarding the same strain of female mice exposed to γ radiation and offer new insights into male mice.

Regarding the influence of sex, we found that females were at a relatively greater risk of lifespan shortening after exposure to γ rays or carbon ions. Again, this result is consistent with the findings from epidemiological studies of atomic-bomb survivors, i.e., that female survivors were at higher risk of developing a solid cancer [40]. In previous animal studies, lifespan analyses using male and female mice irradiated with γ rays, X rays and neutrons show varying results across reports, with some indicating no differences between the sexes [31,32,41,42], possibly related to differences in mouse strain and/or age at exposure. Female B6C3F1 mice exposed to low-dose-rate γ rays exhibited shorter lifespans than their male counterparts [22]. Notably, in previous studies using B6C3F1 female mice [21,24], the induction of liver tumors by radiation exposure was linked to the lifespan shortening, suggesting that they might have caused the lifespan shortening in the present study. Our study is the first to provide data on differences in lifespan between males and females irradiated with carbon ions. It may be difficult to reproduce the data presented here in the same large-scale experiment. However, in the future, biomarkers that closely represent the molecular mechanism of carcinogenesis might serve as surrogates to confirm the validity of the present study in smaller experiments [43].

The relationship between the risk of lifespan shortening and dose within the 0–2 Gy range showed no significant differences between linear and linear-quadratic models. However, the linear-quadratic model provided a better fit for the higher dose range of up to 4.8 Gy for mice exposed to γ rays or carbon ions at 1 week of age. These results fit the latest results obtained for atomic-bomb survivors (1958–2009) [40,44], indicating a linear-quadratic dose response for the entire dose range (0–3 Gy) with a more linear dose-response for the lower dose range (0–1 Gy), which is possibly related to inclusion of more recent data. Future epidemiological studies are expected to include data pertaining to diseases occurring in populations exposed at still earlier ages, which may ultimately help support a stronger fit to the linear-quadratic model. Although high-dose carbon-ion exposure of the order of Gy is not expected during space travel, there is potential for high-dose exposure when administering hypofractionation therapy in carbon-ion radiotherapy. Caution should be exercised, particularly when treating younger patients, and a multiple fractionation protocol is recommended.

Our results show that the RBE of low-LET (~13 keV/µm) carbon ions for lifespan shortening was 1–2, regardless of the age at exposure. This was unexpected given the previously reported RBE (0.2–4.3) on carcinogenesis in specific tissues, which varied depending on age [27,30]. Additionally, the RBEs for lung tumor mortality differed between 1.3 for males and 3.2 for females [29], but the RBEs for intestinal and colon tumor frequencies were similar, at 3.5 for males and 3.3 for females [28]; differences between males and females depended on tissue type. Thus, it is suggested that age susceptibility changed similarly for both γ-ray and carbon-ion irradiation when the effects on multiple tissues are summed up. Although the RBEs for lethality at higher doses than those used in this study were reported as 0.99 in C57BL/6J mice exposed to 7.25 Gy [25] and 1.2 in C3H/He mice exposed to 5.2 to 6.0 Gy [26], these RBEs were similar to those found in this study. Carbon-ion radiotherapy uses the ‘clinical dose’, which is calculated by multiplying the physical carbon-ion dose with the LET-dependent ‘clinical RBE’ based on the potential to kill cells of line HSG [6]. As shown in S2 Fig, when a tumor was irradiated with a clinical dose of 3.6 Gy, the physical dose at the center of the tumor site was 1.49 Gy (i.e., clinical RBE was 2.41 herein), whereas the clinical and physical doses given to the tissue in front of the tumor were 1.69 Gy and 1.26 Gy, respectively (i.e., clinical RBE was 1.34 herein [6]). Of note, the clinical RBE of 1.34 reported by Inaniwa et al. agrees with the RBE range of 1–2 obtained for lifespan shortening in our study. Consistently, both model-based risk prediction studies and retrospective cohort studies of patients with prostate or uterine cervical cancer (most >40 years of age) have shown an equal or lower risk of second cancer after carbon-ion radiotherapy compared with photon radiotherapy [11–14]. Thus, our current results provide fundamental knowledge for understanding the long-term risk in healthy tissue after carbon-ion exposure, considering the age of people at exposure.

Our study has the following limitations. First, the RBEs for lifespan shortening obtained were for animals, which may not truly reflect a human’s sensitivity to radiation-induced morbidity. Observational studies of patients treated with carbon-ion or photon radiotherapy could be used to validate the predictive power of RBE as we report herein. Second, although individual tissues may vary in their sensitivity to radiation dose, age at exposure, and type of radiation, our study did not address tissue-specific susceptibilities. This possibility is currently being investigated by pathological examination. Notably, results on the pathological analysis of limited specific tumor types (e.g., lung tumors, B-cell lymphomas, T-cell lymphomas) as well as molecular biological analyses have also been published [29,45–47]. Third, exposure to carbon-ion radiation is assumed to include whole-body exposure from repeated spaceflights and conventional carbon-ion radiotherapy utilizes partial-body, fractionated irradiation. In contrast, the present study utilized a single round of whole-body irradiation, which facilitated the estimation of mortality risks related to all organs. The use of single irradiation also has merit for understanding age dependence because fractionated irradiation requires a longer time frame, which reduce the accuracy of estimating age dependence in mice experiments. On the other hand, interaction between the irradiated organs, which is unlikely in actual human therapy, cannot be excluded in a whole-body irradiation experiment in mice. In human therapy, the various effects would be reduced in the case of fractionated and partial irradiation compared to single whole-body irradiation, according to previous reports [48]. Fourth, more than 90% of mice herein were terminated at a humane endpoint, whereas historical studies often used natural death as the study endpoint. Of note, two historical studies terminating the B6C3F1 mice at their natural death [21,22] and the recent two studies adopting humane endpoints in the same strain (Shang et al. and the present study) [23] have shown similar average lifespans among the nontreated animal groups. This indicates that the set of humane endpoints adopted herein is a very good surrogate for animal death. Thus, the difference in the endpoints is not likely to noticeably impact the lifespan data.

## Conclusion

The present study used mice to demonstrate the lifespan-shortening effect of low-LET carbon ions, a type of particle radiation to which humans may be exposed as cosmic radiation and as radiation passing through healthy tissue during carbon ion radiotherapy. The results showed that the risk of lifespan shortening associated with both gamma rays and low-LET carbon ion exposure varied with age and sex, but the RBE of low-LET carbon ions for lifespan shortening did not. This is the first report of the RBE of low-LET carbon ions concerning lifespan shortening of mice of both sexes and at various ages. These findings provide essential data for assessing the impacts of gamma rays and low-LET carbon ion exposure.

## Supporting information

S1 Fig Irradiation container used for γ-ray or carbon ion irradiation. A. Mice were placed in 12 compartments and irradiated inside the Gammacell. B. The irradiation container was composed of an inner container and an outer container. The inner container was for holding mice. By changing the partition (half, one-third, one-quarter) according to the size of the mouse body, it was possible to test 2–4 mice. The external container was sealed and maintained SPF status during transport and irradiation outside the SPF area. Sterile filters were set on two tubes connected to the air inlet and outlet of the container, and sterile air was circulated using a pump. The panel at the back of the outer container was designed so that the center of the inner container became the center of irradiation when set on the irradiation table. C. During irradiation, the dose and dose rate could be set for each irradiation container, and up to 10 containers could be irradiated.
(TIFF)

S2 Fig Dose distribution when irradiating the center of the tumor with a clinical dose of 3.6 Gy. The graph was created based on data reported by Inaniwa et al. (2015) [6]. The values in terms of gray (Gy) were provided by personal communication with Dr. Inaniwa.
(TIFF)
